# Serum Vascular Adhesion Protein-1 Predicts End-Stage Renal Disease in Patients with Type 2 Diabetes

**DOI:** 10.1371/journal.pone.0147981

**Published:** 2016-02-04

**Authors:** Hung-Yuan Li, Hung-An Lin, Feng-Jung Nien, Vin-Cent Wu, Yi-Der Jiang, Tien-Jyun Chang, Hsien-Li Kao, Mao-Shin Lin, Jung-Nan Wei, Cheng-Hsin Lin, Shyang-Rong Shih, Chi-Sheng Hung, Lee-Ming Chuang

**Affiliations:** 1 Department of Internal Medicine, National Taiwan University Hospital, Taipei, Taiwan; 2 Lo-Sheng Sanatorium and Hospital, Ministry of Health and Welfare, Taipei, Taiwan; 3 Department of Internal Medicine, National Taiwan University Hospital Yun-Lin Branch, Yun-Lin, Taiwan; 4 Graduate Institute of Clinical Medicine, Medical College, National Taiwan University, Taipei, Taiwan; 5 Chia Nan University of Pharmacy and Science, Tainan, Taiwan; 6 Division of Cardiovascular Surgery, Department of Surgery, Taipei Medical University-Wan Fang Hospital, Taipei, Taiwan; 7 Graduate Institute of Preventive Medicine, National Taiwan University School of Public Health, Taipei, Taiwan; University of Sao Paulo Medical School, BRAZIL

## Abstract

**Background:**

Diabetes is the leading cause of end-stage renal disease (ESRD) worldwide. Vascular adhesion protein-1 (VAP-1) participates in inflammation and catalyzes the deamination of primary amines into aldehydes, hydrogen peroxide, and ammonia, both of which are involved in the pathogenesis of diabetic complications. We have shown that serum VAP-1 is higher in patients with diabetes and in patients with chronic kidney disease (CKD), and can predict cardiovascular mortality in subjects with diabetes. In this study, we investigated if serum VAP-1 can predict ESRD in diabetic subjects.

**Methods:**

In this prospective cohort study, a total of 604 type 2 diabetic subjects were enrolled between 1996 to 2003 at National Taiwan University Hospital, Taiwan, and were followed for a median of 12.36 years. The development of ESRD was ascertained by linking our database with the nationally comprehensive Taiwan Society Nephrology registry. Serum VAP-1 concentrations at enrollment were measured by time-resolved immunofluorometric assay.

**Results:**

Subjects with serum VAP-1 in the highest tertile had the highest incidence of ESRD (p<0.001). Every 1-SD increase in serum VAP-1 was associated with a hazard ratio of 1.55 (95%CI 1.12–2.14, p<0.01) for the risk of ESRD, adjusted for smoking, history of cardiovascular disease, body mass index, hypertension, HbA1c, duration of diabetes, total cholesterol, use of statins, ankle-brachial index, estimated GFR, and proteinuria. We developed a risk score comprising serum VAP-1, HbA1c, estimated GFR, and proteinuria, which could predict ESRD with good performance (area under the ROC curve = 0.9406, 95%CI 0.8871–0.9941, sensitivity = 77.3%, and specificity = 92.8%). We also developed an algorithm based on the stage of CKD and a risk score including serum VAP-1, which can stratify these subjects into 3 categories with an ESRD risk of 0.101%/year, 0.131%/year, and 2.427%/year, respectively.

**Conclusions:**

In conclusion, serum VAP-1 can predict ESRD and is a useful biomarker to improve risk stratification in type 2 diabetic subjects.

## Introduction

More than 371 million people had diabetes mellitus (DM) in 2012, and this number is predicted to increase to 559 million in 2030.[1] Diabetes results in increased mortality and morbidity. Although the mortality rate in diabetic subjects has decreased in Taiwan in the past 10 years,[2] the prevalence of diabetic nephropathy and end-stage renal disease (ESRD) is still increasing.[3] Indeed, diabetes is the leading cause of ESRD worldwide.[4] The risk of ESRD can be estimated by the chronic kidney disease (CKD) stage; however, the prediction is not accurate and could be further improved.

Vascular adhesion protein-1 (VAP-1), encoded by the *AOC3* gene, is found in the serum, adipocytes, smooth muscle, and endothelium.[5–8] VAP-1 is rapidly translocated to the membrane of endothelial cells during an inflammatory process.[9, 10] VAP-1 participates in leukocyte trafficking, extravasation, and recruitment steps in inflammation.[11] VAP-1 has an enzymatic activity and is also called primary amine oxidase or semicarbazide-sensitive amine oxidase (SSAO), because it can catalyze the breakdown of primary amines and its activity can be inhibited by semicarbazide.[12] SSAO catalyzes oxidative deamination of primary amines to produce aldehydes, hydrogen peroxide, and ammonia. Aldehydes enhance non-enzymatic glycation of proteins.[13] Hydrogen peroxide is a source of reactive oxygen species that can cause cellular damage. Along with aldehyde, hydrogen peroxide can cross-link proteins and form advanced glycation end-products (AGEs).[14] These toxic end-products of SSAO may lead to the development of diabetes and diabetic complications such as retinopathy, nephropathy, neuropathy, and atherosclerosis.[15] In animals, VAP-1 overexpression results in glomerulosclerosis, whereas SSAO inhibition reduces the severity of diabetic nephropathy.[15, 16]

VAP-1 has a soluble form whose concentration could be regulated by tumor necrosis factor.[8] Many studies, including a report published by our group, have shown that circulating VAP-1 is increased in diabetic subjects.[17] We have demonstrated that serum VAP-1 can predict cardiovascular and all-cause mortality in diabetic patients.[18] In addition, in our previous report, we have shown that serum VAP-1 is associated with the severity of albuminuria positively and estimated GFR negatively.[19] In this cohort study, we investigated if serum VAP-1 can predict the development of ESRD in diabetic patients. We also explored the use of serum VAP-1 clinically to estimate the risk of ESRD.

## Materials and Methods

### Subjects

We performed a prospective cohort study.[18] Between July 1996 and June 2003, all subjects with type 2 diabetes who were regularly followed-up at outpatient clinics at the Division of Endocrinology and Metabolism, National Taiwan University Hospital, Taipei, Taiwan, were invited consecutively to participate in the study. Subjects who had developed ESRD before the follow-up were excluded from the study. Written informed consent was obtained from each subject, and the study protocol was reviewed and approved by the Institutional Review Board of the National Taiwan University Hospital.

Each subject was interviewed and underwent a physical examination by physicians. The history of cardiovascular disease was confirmed from the medical records at admission. Venous blood sampling was performed after overnight fasting for the determination of plasma glucose, hemoglobin A1c (HbA1c), serum total cholesterol, triglyceride, and creatinine by using an automatic analyzer (Toshiba TBA 120FR; Toshiba Medical Systems Co., Tokyo, Japan). Estimated GFRs were calculated with the Chronic Kidney Disease Epidemiology (CKD-EPI) Collaboration equation.[20] The CKD-EPI equation, expressed as a single equation, is as follows: GFR = 141 × min(Scr/κ, 1)^α^ max(Scr/κ, 1)^-1.209^ × 0.993^Age^ × 1.018 [if female] × 1.159 [if black]; where Scr is serum creatinine, κ is 0.7 for female and 0.9 for male patients, α is -0.329 for female and -0.411 for male patients, min indicates the minimum of Scr/κ or 1, and max indicates the maximum of Scr/κ or 1.

Serum samples were stored at -80°C before the measurement of VAP-1. Spot urine samples were collected to determine the presence of proteinuria by performing reflectance colorimetry (Arkray AX4280; Japan). The ankle-brachial index (ABI) was measured as the ratio of the systolic blood pressure of the posterior tibial artery or dorsalis pedis and the brachial artery. An ABI greater than 1.3 or lesser than 0.9 was defined as abnormal.[21]

To ascertain the status of ESRD, we linked our database with the nationally comprehensive Taiwan Society Nephrology registry, which receives the data reports of all dialysis patients in Taiwan every 3 months till May 1, 2011.[22]

### Serum VAP-1 measurement

Serum VAP-1 and its SSAO activity are quite stable. When stored properly at -70°C, it has been shown to remain intact even after 2 years.[23] Serum VAP-1 was measured using a time-resolved immunofluorometric assay as stated previously.[17–19, 24] Briefly, the assay utilized a biotin-conjugated monoclonal anti-human VAP-1 antibody (Biotie Therapies Corp., Finland) as a capture antibody on a streptavidin-coated microtiter plate. Bound serum VAP-1 was detected using a different europium-conjugated anti-human VAP-1 antibody (Biotie Therapies Corp.). The time-resolved fluorescence was measured using a fluorometer (Victor^2^ Multilabel Counter; PerkinElmer Finland Oy) at 615 nm. Serum VAP-1 concentration was quantified on the basis of a reference sample of highly purified human serum VAP-1 (Biovian Ltd). The R^2^ of the standard curves was 0.997–1.000. The intraassay coefficients of variation were 3.7%, 5.2%, and 8.9% for quality control samples with concentrations of 1000, 500, and 100 ng/ml, respectively. The inter-batch coefficients of variation from quality control samples were 4.4–10.2%.

### Statistical analysis

Categorical variables were summarized as the percentage of patients in the subgroup. The distributions of continuous variables were examined by the Shapiro-Wilk test. Continuous variables distributed normally were presented as means and standard deviations (SD). Continuous variables with skewed distribution were analyzed after logarithmic transformation and were presented as medians (interquartile ranges). Student’s *t*-tests, Chi-square tests, and analysis of variance were used to access the differences in clinical characteristics between subjects who developed ESRD and those who did not develop ESRD, and among different subgroups by serum VAP-1 tertile. ESRD in subgroups was estimated by the Kaplan-Meier method and was tested by log-rank test. Multivariate Cox proportional hazard models were applied to estimate the hazard ratios (HRs) of predictors for ESRD. Variables significantly associated with ESRD in univariate Cox proportional hazard models, as well as the clinically important variables, were included in multivariate analyses. We constructed risk scores by using the regression coefficients in the multivariate Cox proportional hazard models. In addition, an algorithm was developed by dividing the subjects into 2 groups according to their CKD stage. We then constructed another risk score to further divide subjects with CKD stage 3–5 into 2 groups. We also calculated the incidence of progression to ESRD for subjects in different subgroups. Area under the ROC curve was used to assess the ability of serum VAP-1 and the risk scores to predict the ESRD during follow-up. They ranged from 0.5 (no predictive ability) to 1 (perfect predictive ability). Both risk scores were validated by the leave-one-out methods.[25] A two-tailed p-value below 0.05 was considered significant. Stata/SE 11.0 for Windows (StataCorp LP, TX) was used for statistical analyses.

## Results

We recruited 604 patients with type 2 diabetes. During a median follow-up period of 12.36 years (interquartile range 11.50–13.99 years), 22 patients developed ESRD. Subjects who developed ESRD showed a higher serum VAP-1 concentrations (median, 941 ng/ml vs. 689 ng/ml, p<0.0001, Table 1). As shown in Table 2, patients with serum VAP-1 in the highest tertile had a higher incidence of ESRD and proteinuria, and lowest percentage of subjects who smoked. Most of them were female patients. They were more likely to have older age, longer duration of diabetes, lower estimated GFR, and higher plasma glucose, HbA1c, and total cholesterol levels than patients with serum VAP-1 in the second tertile and first tertile.

**Table 1:** Baseline characteristics of subjects with type 2 diabetes stratified by end-stage renal disease (ESRD).

	ESRD not developed	ESRD developed	P-value
N (%)	582 (96.4)	22(3.6)	
**Follow-up period (years)**	**13.11 (12.28–14.03)**	**6.29 (3.72–8.86)**	**<0.001**
Age (years)	61.7 ± 9.7	63.0 ± 7.8	0.5
Men (%)	49.1	40.9	0.4
Smoking (%)	16.7	22.7	0.5
**History of cardiovascular disease (%)**	**10.5**	**31.8**	**0.002**
SBP (mmHg)	134 ± 17	139 ± 14	0.2
DBP (mmHg)	79 ± 9	81 ± 10	0.3
**Hypertension drugs (%)**	**32.7**	**59.1**	**0.01**
**Hypertension (%)**	**59.5**	**81.8**	**0.04**
**Duration of diabetes (years)**	**8 (3–14)**	**15.5 (9–25)**	**0.001**
Fasting plasma glucose, mmol/l (mg/dl)	8.3 ± 2.5 (150 ± 45)	8.8 ± 2.6 (159 ± 47)	0.3
Postprandial plasma glucose, mmol/l (mg/dl)	11.9 ± 4.1 (215 ± 74)	11.9 ± 4.4 (216 ± 80)	0.9
**HbA1c (%)(mmol/mol)**	**7.6 ± 1.4 (60 ± 15.3)**	**8.9 ± 1.6 (74 ± 17.5)**	**<0.001**
**Total cholesterol, mmol/l (mg/dl)**	**5.20 ± 1.03 (201 ± 40)**	**5.77 ± 1.11 (223 ± 43)**	**0.01**
**Statins (%)**	**3.44**	**13.64**	**0.01**
**Triglyceride, mmol/l (mg/dl)**	**1.50 (1.06–2.21), 133 (94–196)**	**2.13 (1.33–2.72), 189 (118–241)**	**0.04**
BMI (kg/m^2^)	24.6 ± 3.3	25.6 ± 3.3	0.15
24–27 (%)	31.2	31.8	0.2
≥27 (%)	20.8	36.4	
**Creatinine, μmol/l (mg/dl)**	**79.6 ± 27 (0.9 ± 0.3)**	**150 ± 70 (1.7 ± 0.8)**	**<0.001**
**Estimated GFR (mL/min per 1.73 m**^**2**^**)**	**78.8 ± 19.4**	**45.9 ± 22.1**	**<0.001**
**Proteinuria (%)**	**14.1**	**86.4**	**<0.01**
**ABI < 0.9 or >1.3 (%)**	**6.53**	**27.3**	**<0.01**
**Serum VAP-1 (ng/ml)**	**689 (582–819)**	**941 (849–1024)**	**<0.001**
**By tertile**			**<0.01**
**Middle, 622–779 (%)**	**34.9**	**13.6**	
**Highest, ≥779 (%)**	**31.1**	**81.8**	

Mean ± SD or median values (interquartile ranges) are shown.

Abbreviations: ABI, ankle-brachial index; BMI, body mass index; DBP, diastolic blood pressure; GFR, glomerular filtration rate; SBP, systolic blood pressure; HbA1c: glycated hemoglobin.

**Table 2:** Baseline characteristics stratified by serum vascular adhesion protein-1 (VAP-1) tertile in subjects with type 2 diabetes.

Serum VAP-1 tertile (ng/ml)	<624	624–777	≥777	P-value
N	201	202	201	
**ESRD (%)**	**0.5**	**1.49**	8.96*^†^	**<0.001**
**Follow-up period (years)**	**13.02(10.53–14.02)**	**13.17 (12.60–14.01)**	**13.06**^†^ **(11.78–13.84)**	**0.02**
**Age (years)**	**59.5 ± 9.4**	61.8 ± 9.5*	63.8 ± 9.5*^†^	**<0.001**
**Men (%)**	**56.7**	**48.0**	41.8*	**0.01**
**Smoking (%)**	**21.9**	**17.3**	11.4*	**0.02**
History of cardiovascular disease (%)	12.9	8.9	11.9	0.4
SBP (mmHg)	134 ± 16	135 ± 14	135 ± 19	0.99
DBP (mmHg)	79.5 ± 9.4	78.2 ± 8.8	78.1 ± 8.7	0.2
Hypertension drugs (%)	29.9	35.1	35.8	0.4
Hypertension (%)	59.7	61.9	59.2	0.8
**Duration of diabetes (years)**	**5 (2–11)**	8.5* (4–13)	11*^†^ **(6–18)**	**<0.001**
**Fasting plasma glucose (mmol/l)**	**7.71 ± 1.97**	8.31 ± 2.34*	8.97 ± 2.99*^†^	**<0.001**
**Postprandial plasma glucose (mmol/l)**	**11.23 ± 3.63**	**11.28 ± 3.49**	13.08 ± 4.80*^†^	**<0.001**
**HbA1c (%) (mmol/mol)**	**7.1 ± 1.3 (54 ±14.2)**	7.7 ± 1.3* (61 ±14.2)	8.2 ± 1.6 (66 ±17.5)*^†^	**<0.001**
**Total cholesterol (mmol/l)**	**5.08 ± 1.00**	**5.27 ± 0.99**	5.33 ± 1.09*	**0.049**
Statins (%)	1.99	5.94	3.48	0.11
Triglyceride (mmol/l)	1.51 (1.07–2.27)	1.63 (1.17–2.25)	1.45 (0.97–2.15)	0.47
BMI (kg/m^2^)	24.6 ± 3.0	24.7 ± 3.2	24.5 ± 3.7	0.7
24–27 (%)	33.33	33.17	27.36	0.4
≥27 (%)	17.91	22.28	28.88	
**Creatinine (μmol/l)**	**79.6 (70.7–97.2)**	**79.6 (70.7–88.4)**	79.6*^†^ **(70.7–106.1)**	**0.003**
**Estimated GFR (mL/min per 1.73 m**^**2**^**)**	**82.4 ± 18**	**78.8 ± 19**	71.6 ± 23*^†^	**<0.001**
**Proteinuria (%)**	**10.3**	**11.2**	28.6*^†^	**<0.01**
ABI < 0.9 or > 1.3 (%)	5.53	8.25	8.04	0.5

Mean ± SD or median values (interquartile ranges) are shown.

* p < 0.05 vs. first tertile (serum VAP-1 <624 ng/ml)

† p < 0.05 vs. second tertile (serum VAP-1 624–777 ng/ml)

Abbreviations: ABI, ankle-brachial index; BMI, body mass index; DBP, diastolic blood pressure; GFR, glomerular filtration rate; SBP, systolic blood pressure; HbA1c: glycated hemoglobin

**Fig 1:**
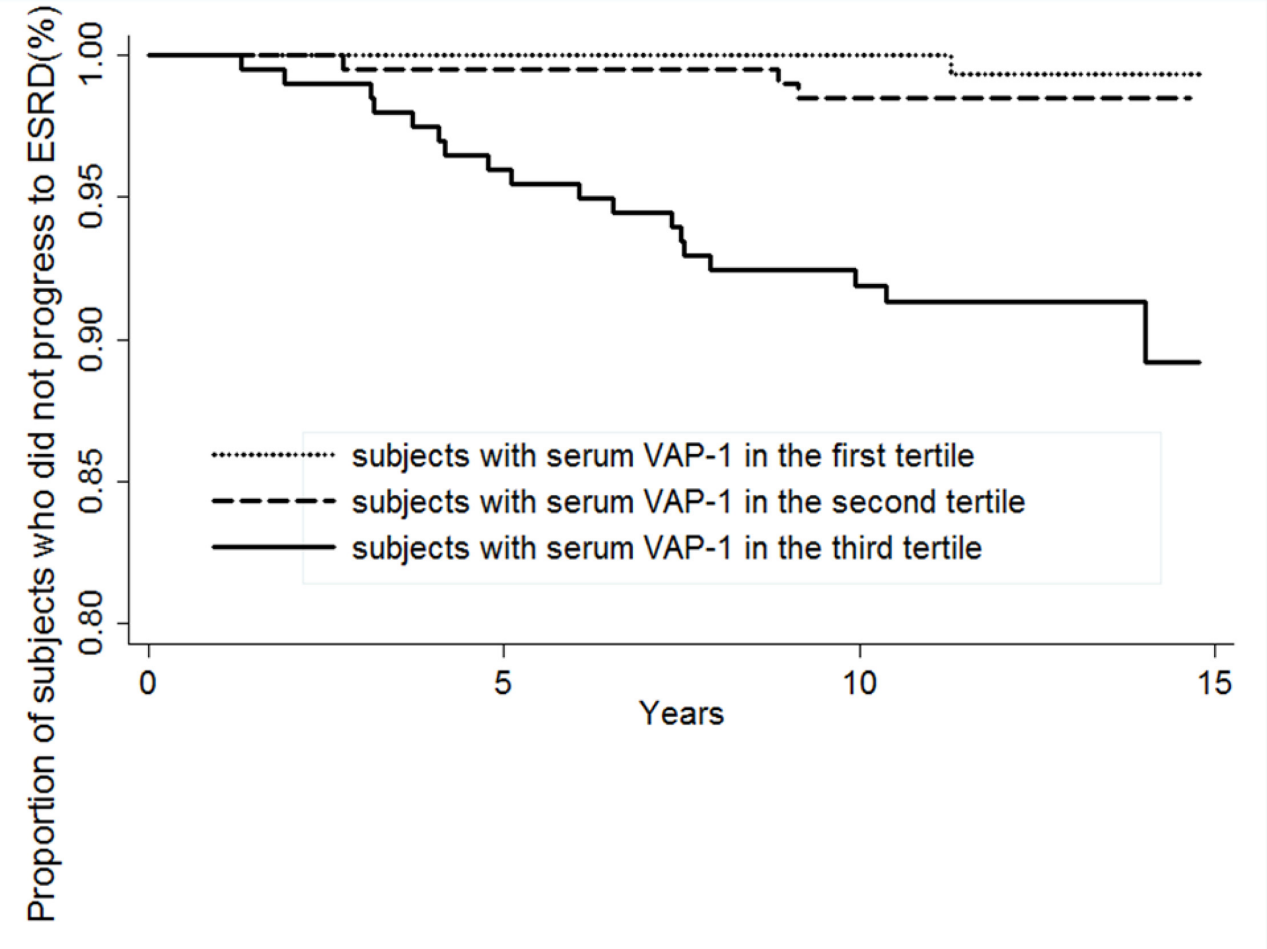
Kaplan-Meier survival curves by tertile of serum VAP-1 concentrations. Dotted line = subjects with serum VAP-1 in the first tertile; dashed line = subjects with serum VAP-1 in the second tertile; solid line = subjects with serum VAP-1 in the third tertile. P < 0.001 among the subgroups by tertile.

Fig 1 shows that subjects with serum VAP-1 in the highest tertile had the highest incidence of ESRD (p < 0.001), as shown by Kaplan-Meier survival curves. The area under the ROC for serum VAP-1 to predict ESRD was 0.8214 (95% CI 0.73048–0.91229). As shown in model 1 and 2 in Table 3, higher serum VAP-1 levels were associated with increased risk of ESRD (HR of every 1 SD increase in serum VAP-1 = 1.55, 95% CI 1.12–2.14, p < 0.05), after adjusting for smoking, history of cardiovascular disease, BMI, hypertension, HbA1c, duration of DM, total cholesterol levels, use of statins, abnormal ABI, estimated GFR, and proteinuria.

**Table 3:** Hazard ratios (95% confidence intervals) of end-stage renal disease in subjects with type 2 diabetes.

	Crude	Model 1	Model 2
**Every 1 SD increase in serum VAP-1**	2.10* (1.69–2.61)	**1.52**^**†**^ **(1.14–2.03)**	**1.55**^**†**^ **(1.12–2.14)**
Age (year)	1.01(0.97–1.06)		
Female gender	1.42(0.61–3.32)		
Smoking	1.43(0.53–3.86)		2.03(0.65–6.37)
History of cardiovascular disease	**4.26**^**‡**^**(1.73–10.53)**		1.19(0.37–3.92)
BMI (kg/m^2^)			
<24	1		1
24–27	1.54 (0.54–4.39)		3.74§(0.88–16.60)
≥27	**2.61**^**§**^ **(0.95–7.20)**		0.96(0.25–3.76)
P for trend	.056		.426
Hypertension	**2.97**^**‡**^ **(1.01–8.78)**		1.27(0.38–4.26)
HbA1c (%)	1.55*(1.27–1.89)	**1.39**^**†**^ **(1.03–1.87)**	1.39^**§**^(1.00–1.95)
DM duration (years)	1.08*(1.04–1.12)		1.00(0.95–1.06)
Total cholesterol (mmol/l)	**1.58**^**†**^**(1.12–2.22)**		1.00(0.64–1.56)
Statins	**4.46**^**‡**^**(1.32–15.08)**	**5.61**^**†**^ **(1.57–20.13)**	**5.45**^**‡**^ **(1.34–22.21)**
ABI < 0.9 or > 1.3	**5.23**^**†**^**(2.05–13.38)**		1.57(0.53–4.62)
Estimated GFR (mL/min per 1.73 m^2^)	0.93*(0.91–0.95)	0.94* (0.92–0.97)	0.95* (0.92–0.98)
Proteinuria	35.94*(10.63–121.54)	**8.54**^**‡**^ **(2.21–32.94)**	**8.30‡ (2.09–32.99)**

1 SD of serum VAP-1 = 196.4 ng/ml

* p < 0.001

† p < 0.01

‡ p < 0.05

§ 0.05< p < 0.10

Model 1 included serum VAP-1, HbA1c, use of statins, estimated GFR, and proteinuria.

Model 2 included serum VAP-1, smoking, history of cardiovascular disease, BMI, hypertension, HbA1c, DM duration, total cholesterol, use of statins, ABI < 0.9 or > 1.3, estimated GFR, and proteinuria.

Abbreviations: ABI, ankle-brachial index; BMI, body mass index; DBP, diastolic blood pressure; GFR, glomerular filtration rate; SBP, systolic blood pressure; HbA1c: glycated hemoglobin; DM: diabetes mellitus; SD: standard deviation.

**Fig 2:**
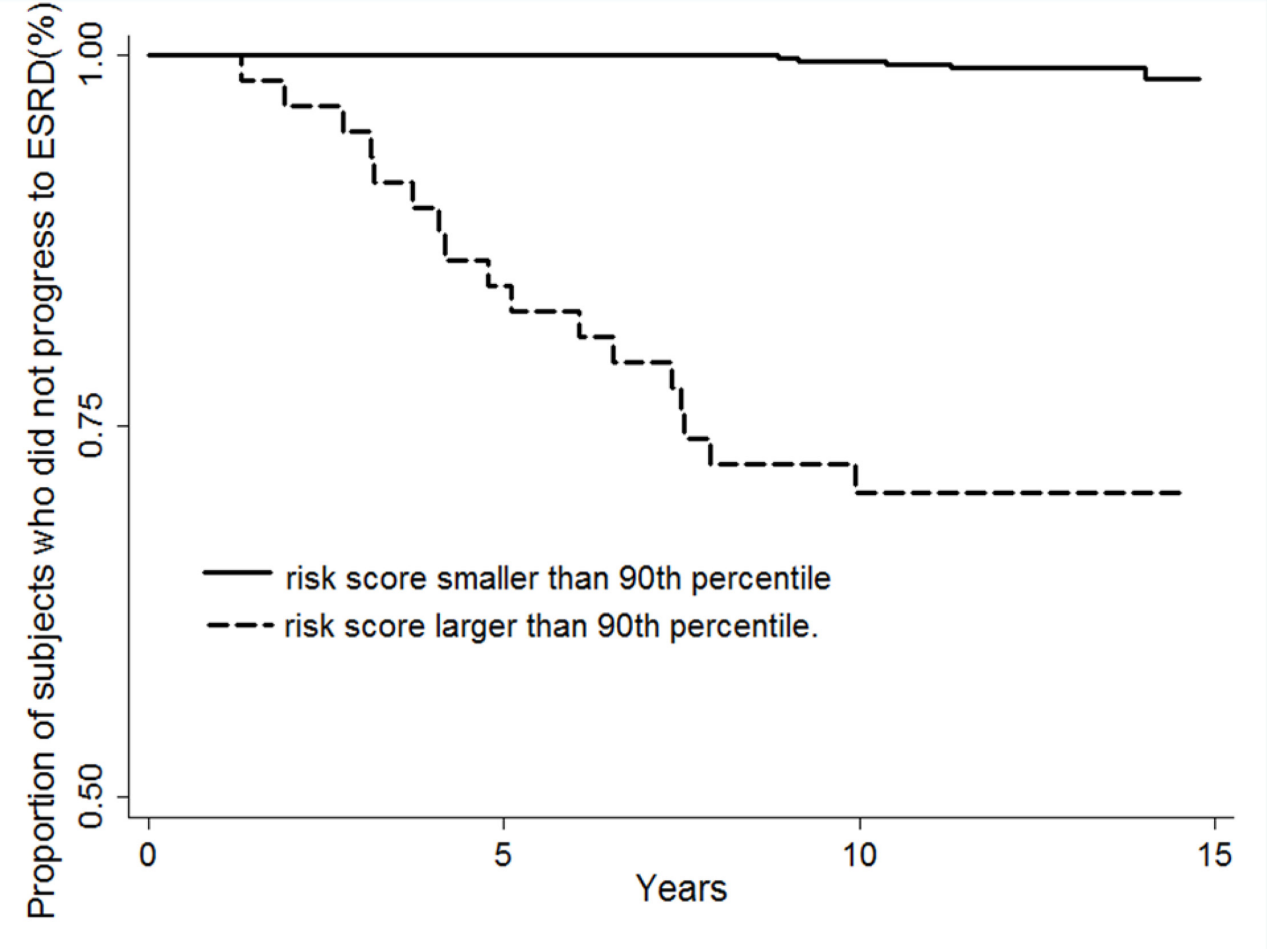
Kaplan-Meier survival curves by risk score for end-stage renal disease in subjects with type 2 diabetes. Risk score = 0.0020656 * serum VAP-1(ng/ml) + 0.3110558 * HbA1c(%) - 0.0562534 * estimated GFR(ml/min per 1.73 m^2^) + 2.142356 if proteinuria was present. Dashed line = risk score larger than 90^th^ percentile; solid line = risk score smaller than 90^th^ percentile. P < 0.0001 between high-risk and low-risk subgroups. Performance of the risk score: area under ROC = 0.9406 (95% CI 0.8871–0.9941), sensitivity = 77.3%, specificity = 92.8%. Internal validation by the leave-one-out method showed that the sensitivity was 86.4% (95% CI 65.1–97.1%) and the specificity was 89.9% (95% CI 87.1–92.3%).

Subsequently, we constructed a risk score to predict ESRD by using variables significantly predicted ESRD in Table 3. At that time, the potency of statins was weaker and the threshold of cholesterol concentrations to start statins was higher. Therefore, the use of statins was a marker of hypercholesterolemia. Indeed, subjects who used statins had higher plasma total cholesterol concentrations (245 vs. 200 mg/dl, p < 0.001). Therefore, the risk score composed of serum VAP-1, HbA1c, estimated GFR and proteinuria, but did not include the use of statins. As shown in Fig 2, subjects with risk scores greater than the 90^th^ percentile showed higher risk for ESRD (p < 0.001). The performance of the risk score to predict ESRD was good. The area under the ROC for the risk score to predict ESRD was 0.9406 (95% CI 0.8871–0.9941). The sensitivity was 77.3%, and the specificity was 92.8%. We validated the risk score internally by the leave-one-out method, which showed that the sensitivity and specificity were 86.4% (95% CI 65.1–97.1%) and 89.9% (95% CI 87.1–92.3%), respectively. Table 4 summarized the area under the ROC for different variables to predict ESRD. The risk score and the linear combination of estimated GFR and proteinuria showed significantly higher area under the ROC than serum VAP-1 alone, estimated GFR alone, and proteinuria alone (all p<0.05). Besides, the area under the ROC of the risk score was higher than that of the combination of estimated GFR and proteinuria (0.9406 *vs*. 0.9225, p = 0.0260).

**Table 4:** Comparisons of variables in the prediction of end-stage renal disease in subjects with type 2 diabetes.

Predictors	Area under the ROC (95% CI)	P values vs. serum VAP-1	P values vs. risk score
All subjects			
Serum VAP-1	0.8214 (0.7305–0.9123)		**0.0002**
Estimated GFR	0.8648 (0.7764–0.9533)	0.4437	**0.0353**
Proteinuria	0.8614 (0.7866–0.9362)	0.3310	**0.0006**
Risk score*	0.9406 (0.8871–0.9941)	**0.0002**	
Estimated GFR + proteinuria†	0.9225 (0.8626–0.9824)	**0.0060**	**0.0260**
In subjects with CKD stage 3–5			
Serum VAP-1	0.7855 (0.6745–0.8966)		**0.0113**
Estimated GFR	0.8006 (0.6873–0.9139)	0.8592	0.0545
Proteinuria	0.8004 (0.7237–0.8772)	0.7468	**<0.0001**
Risk score‡	0.9053 (0.8249–0.9858)	**0.0113**	
Estimated GFR + proteinuria§	0.8719 (0.7951–0.9488)	0.1565	0.1846

* Risk score = 0.0020656 * serum VAP-1(ng/ml) + 0.3110558 * HbA1c(%) - 0.0562534 * estimated GFR(ml/min per 1.73 m^2^)+ 2.142356 if proteinuria was present.

† linear combination = -0.0466015*eGFR_CKD_EPI + 2.681692 if proteinuria was present.

‡ Risk score = 0.0020192 * serum VAP-1(ng/ml) + 0.3105567 * HbA1c(%) - 0.0730014 * estimated GFR(mL/min per 1.73 m^2^) + 2.169204 if proteinuria was present.

§linear combination = -0.0466015*eGFR_CKD_EPI + 2.681692 if proteinuria was present.

**Fig 3:**
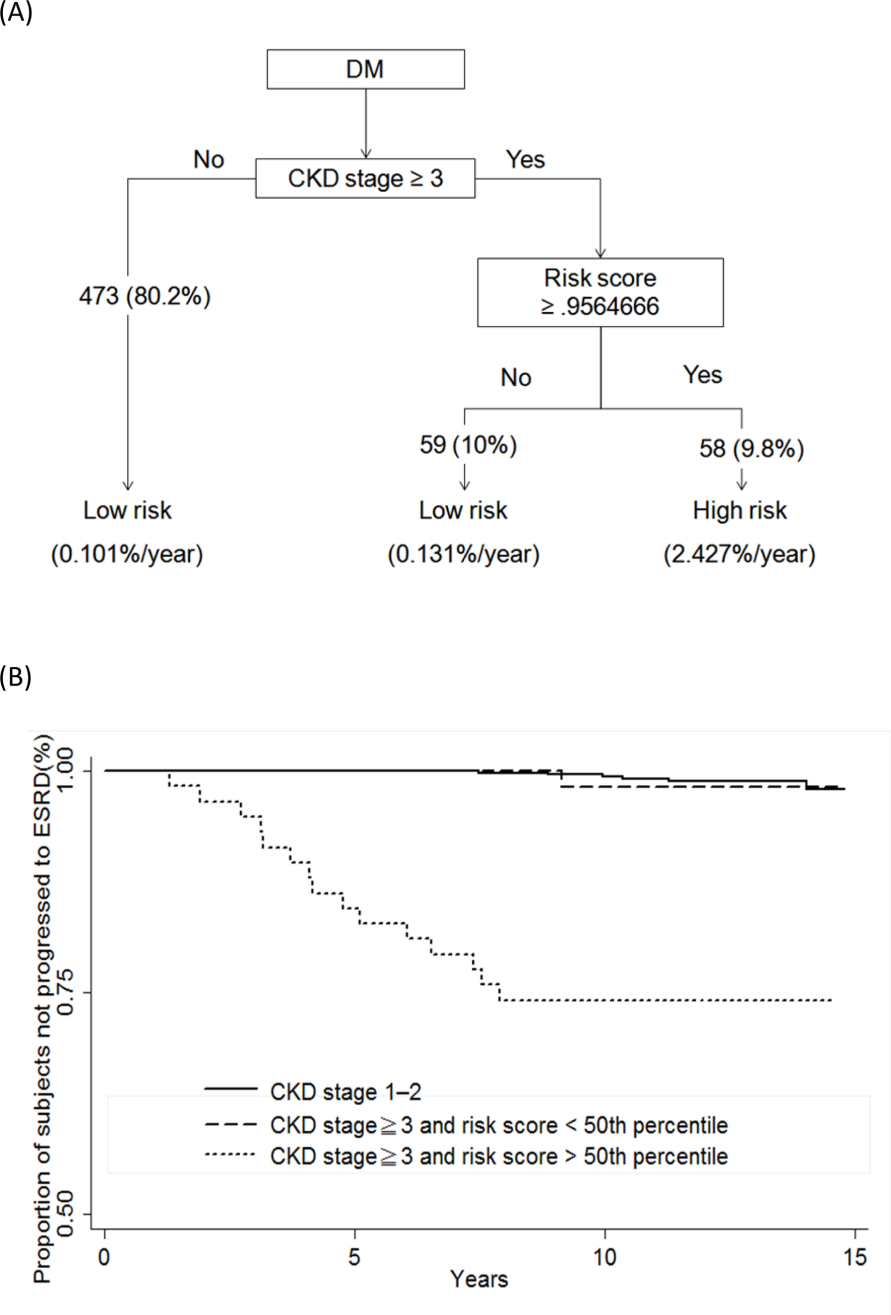
(A) Algorithm for risk of dialysis in subjects with type 2 diabetes. (B) Kaplan-Meier survival curves by risk score and chronic kidney disease (CKD) stage for end-stage renal disease in subjects with type 2 diabetes. Risk score = 0.0020192 * serum VAP-1(ng/ml) + 0.3105567 * HbA1c(%) - 0.0730014 * estimated GFR(mL/min per 1.73 m^2^) + 2.169204 if proteinuria was present. Solid line = risk score larger than 50^th^ percentile; dashed line = risk score smaller than 50^th^ percentile; dotted line = subjects with type 2 diabetes and CKD stage 1–2. P < 0.0001 among the 3 subgroups. Performance of the risk score in subjects with CKD stage 3–5: sensitivity = 93.8% (68.8–99.8), specificity = 88.1% (80.2–93.7) by the leave-one-out method.

Because the CKD stage is an important predictor for ESRD, we developed an algorithm that considered the stage of CKD. As shown in Fig 3A, subjects without CKD or with CKD stage 1 and 2 had a risk of ESRD of 0.101%/year. Serum VAP-1 can predict ESRD in subjects with CKD stage 3–5 (HR of every 1 SD increase in serum VAP-1 = 1.54, 95% CI 1.053–2.26, p < 0.05). A risk score was constructed for subjects with CKD stage 3–5, composed of serum VAP-1, HbA1c, estimated GFR, and proteinuria. The risk score re-classified half of the subjects with CKD stage 3–5 into the low-risk group (0.131%/year). Subjects with CKD stage 3–5 and high-risk scores had a higher risk of ESRD (2.427%/year) than those in the other 2 subgroups (Fig 3B). In subjects with CKD stage 3–5, the area under the ROC of the risk score was significantly higher than serum VAP-1 alone (p = 0.0113), estimated GFR alone (p = 0.0545), and proteinuria alone (p<0.0001) (Table 4). However, although the area under the ROC of the combination of estimated GFR and proteinuria was numerically higher than that of serum VAP-1 alone, it did not reach statistical significance (0.8719 *vs*. 0.7855, p = 0.1565). Similarly, the risk score was numerically higher than that of the linear combination of estimated GFR and proteinuria, but it did not reach statistical significance (0.9053 *vs*. 0.8719, p = 0.1846).

## Discussion

To the best of our knowledge, this is the first study showing that serum VAP-1 can predict the development of ESRD in subjects with type 2 diabetes. We constructed a risk score with HbA1c, estimated GFR, proteinuria, and serum VAP-1, which predicted ESRD with good performance. We also developed an algorithm that can stratify the risk of ESRD by using information of the CKD stage and another risk score.

DM results in macro- and micro-vascular complications. Low-grade inflammation may be one of the mechanisms for diabetic complications.[26] Hyperglycemia induces excessive production of reactive oxygen species, which activates various pathways such as the polyol pathway, formation of AGEs, protein kinase C pathway, and hexosamine pathway. These pathways then activate and propagate inflammatory processes, which can result in end-organ damage. For example, C-reactive protein has been shown to cause vascular injury by inducing the expression of various adhesion molecules, decreasing endothelial nitric oxide production, and increasing reactive oxygen species.[27] Indeed, many inflammatory markers, including C-reactive protein, have been reported to predict the development of diabetic complications in human subjects.[28, 29]

Elevated serum VAP-1 is associated with acute and chronic hyperglycemia in human subjects, as shown in our previous report.[17] Serum VAP-1 is increased 30 min after oral ingestion of glucose water during an oral glucose tolerance test. Subjects with DM have higher serum VAP-1 than subjects without DM. The higher the HbA1c, the higher the serum VAP-1 concentrations. Endothelial VAP-1 plays a role in leukocyte adhesion, transmigration, and recruitment.[11] In addition, its SSAO activity catalyzes the generation of toxic end-products, including aldehyde, hydrogen peroxide, and ammonia. Aldehydes, in the presence of oxidative stress and hyperglycemia, results in the generation of AGEs.[26] AGEs can regulate gene transcription, modify proteins and extracellular matrix, and induce inflammation, through AGE itself or through binding to its receptor. Hydrogen peroxide is a source of oxidative stress, which results in cell damage through many pathways.[30] Indeed, these end-products have been shown to cause cellular injury, resulting in diabetic complications such as atherosclerosis. [26, 30] Inhibition of SSAO activity results in reduced production of AGEs, LDL oxidation, and endothelial injury.[13] Taken together, these results suggest that VAP-1 is involved in the development of diabetic complications through participation in inflammation and production of toxic metabolites.

Many studies have demonstrated a significant relationship between pro-inflammatory cytokines and the pathological changes of nephrons, such as increased endothelial permeability, proliferation of mesangial cells and matrix, and glomerular microcirculation abnormalities.[29] VAP-1 participates in inflammation; this could be one of the reasons why serum VAP-1 could predict DM nephropathy in the present study. Furthermore, VAP-1 may be involved in diabetic nephropathy through the generation of toxic SSAO metabolites. We have shown that serum VAP-1 is a source of systemic oxidative stress and AGEs in humans.[24] In mice, these SSAO end-products accumulate and show the highest concentrations in the kidneys, as measured by the ingestion of C^14^-methylamine.[31] Indeed, mice over-expressing VAP-1 in their endothelium show increased expression of AGE receptors and develop glomerulosclerosis.[15] Inhibition of SSAO in diabetic mice reduces the severity of diabetic nephropathy.[13] Endothelial VAP-1/SSAO is a source of serum VAP-1/SSAO.[5] In humans, elevated serum VAP-1 is associated with the amount of micro-albuminuria and extent of reduction in GFR.[19] In the present study, elevated serum VAP-1 precedes and is associated with the development of ESRD in diabetic patients. All these data suggest that it is reasonable to develop drugs targeting SSAO as they may prove to be effective at preventing diabetic nephropathy in humans.

There are several reports that demonstrate an important role for VAP-1 in diabetic complications other than nephropathy. VAP-1 expression is increased in the vitreous fluid in patients with proliferative diabetes retinopathy.[32] Serum VAP-1 is also associated with carotid intima-medial thickness in diabetic macrovascular complications as previously reported by our group[24] and confirmed in another report.[33] We have also demonstrated that serum VAP-1 can predict CV mortality and all-cause mortality in subjects with type 2 diabetes.[18]

Our findings have a number of clinical implications. Firstly, we propose that 2 strategies can stratify the risk of ESRD in the future: risk score and algorithm. Both strategies showed better prediction of ESRD than estimated GFR or proteinuria in subjects with type 2 diabetes. Using these 2 strategies, physicians can estimate the risk of ESRD more accurately and resources can be saved and given to patients with higher risk. In addition, more intensive care can be delivered to the high-risk patients. There are numerous studies looking for predictors for the progression of CKD or ESRD aside from estimated GFR and proteinuria, including age, sex, ethnicity, hypertension, diabetes, dyslipidemia, hyperuricemia, hyperphosphatemia, and some novel biomarkers such as fibroblast growth factor 23 (FGF23), and ceruloplasmin.[34] FGF23 is highly expressed in bone. When FGF23 is secreted, it binds to a complex with αKlotho in kidney and augments urinary phosphate excretion.[35, 36] Blood FGF23 is elevated in subjects with CKD or ESRD,[36] and has been shown to predict ESRD independently in subjects with CKD stage 2–3[37] or CKD stage 4–5.[38] Ceruloplasmin, a copper-carrying metalloenzyme, can act as an anti-oxidant or a pro-oxidant in different conditions and is an acute phase protein.[39] Recently, serum ceruloplasmin has been reported to predict progression of diabetic nephropathy.[40] Besides, increased urinary excretion of ceruloplasmin is also predictive for the development of microalbuminuria in patients with type 2 diabetes.[41] However, since blood FGF23 or ceruloplasmin is not measured in this study, comparison for their prediction ability with serum VAP-1 cannot be done. Another clinical implication of our findings is that inhibitors of SSAO or an antibody against VAP-1 may reduce the risk or slows down the progression of diabetic nephropathy.[42]

The strength of this study is the 100% follow-up rate and a long follow-up period. However, this study has some limitations. First, our population was composed of Chinese Han. Whether the finding is similar in other ethnic group should be explored. Second, although the elevation of serum VAP-1 was observed before the development of ESRD, a detailed study on the molecular mechanism by which VAP-1 expression results in diabetic nephropathy should be investigated in future studies.

In conclusion, we found that serum VAP-1 can predict the development of ESRD in subjects with type 2 diabetes for the first time. The risk score and the algorithm using serum VAP-1 can be used to stratify the risk of ESRD in the future.

## References

[1] IDF Diabetes Atlas. 6th ed. Brussels, Belgium: International Diabetes Federation; 2013. 159 p.

[2] Li HY, Jiang YD, Chang CH, Chung CH, Lin BJ, Chuang LM. Mortality trends in patients with diabetes in Taiwan: a nationwide survey in 2000–2009. Journal of the Formosan Medical Association = Taiwan yi zhi. 2012;111(11):645–50. Epub 2012/12/12. 10.1016/j.jfma.2012.09.013 . 23217600

[3] Huang YY, Lin KD, Jiang YD, Chang CH, Chung CH, Chuang LM, et al Diabetes-related kidney, eye, and foot disease in Taiwan: an analysis of the nationwide data for 2000–2009. Journal of the Formosan Medical Association = Taiwan yi zhi. 2012;111(11):637–44. Epub 2012/12/12. 10.1016/j.jfma.2012.09.006 . 23217599

[4] Guariguata L. By the numbers: New estimates from the IDF Diabetes Atlas Update for 2012. Diabetes Res Clin Pract. 2012;98:524–5. 10.1016/j.diabres.2012.11.006 23217268

[5] Stolen CM, Yegutkin GG, Kurkijarvi R, Bono P, Alitalo K, Jalkanen S. Origins of serum semicarbazide-sensitive amine oxidase. Circ Res. 2004;95:50–7. 15178639 10.1161/01.RES.0000134630.68877.2F

[6] Lewinsohn R. Amine oxidase in human blood vessels and non-vascular smooth muscle. J Pharm Pharmacol. 1981;33(9):569–75. Epub 1981/09/01. . 6117634 10.1111/j.2042-7158.1981.tb13868.x

[7] Salmi M, Kalimo K, Jalkanen S. Induction and function of vascular adhesion protein-1 at sites of inflammation. The Journal of experimental medicine. 1993;178(6):2255–60. Epub 1993/12/01. 8245796 10.1084/jem.178.6.2255 PMC2191278

[8] Pannecoeck R, Serruys D, Benmeridja L, Delanghe JR, Geel N, Speeckaert R, et al Vascular adhesion protein-1: Role in human pathology and application as a biomarker. Critical reviews in clinical laboratory sciences. 2015;52(6):284–300. Epub 2015/08/20. 10.3109/10408363.2015.1050714 . 26287391

[9] Abella A, Garcia-Vicente S, Viguerie N, Ros-Baro A, Camps M, Palacin M, et al Adipocytes release a soluble form of VAP-1/SSAO by a metalloprotease-dependent process and in a regulated manner. Diabetologia. 2004;47(3):429–38. Epub 2004/02/18. 10.1007/s00125-004-1346-2 . 14968297

[10] Sallisalmi M, Tenhunen J, Yang R, Oksala N, Pettila V. Vascular adhesion protein-1 and syndecan-1 in septic shock. Acta Anaesthesiol Scand. 2012;56:316–22. 10.1111/j.1399-6576.2011.02578.x 22150439

[11] Koskinen K, Vainio PJ, Smith DJ, Pihlavisto M, Yla-Herttuala S, Jalkanen S, et al Granulocyte transmigration through the endothelium is regulated by the oxidase activity of vascular adhesion protein-1 (VAP-1). Blood. 2004;103(9):3388–95. Epub 2004/01/17. 10.1182/blood-2003-09-3275 . 14726375

[12] Jalkanen S, Salmi M. VAP-1 and CD73, endothelial cell surface enzymes in leukocyte extravasation. Arterioscler Thromb Vasc Biol. 2008;28:18–26. 17962625 10.1161/ATVBAHA.107.153130

[13] Yu PH, Zuo DM. Aminoguanidine inhibits semicarbazide-sensitive amine oxidase activity: implications for advanced glycation and diabetic complications Diabetologia 1997;40:1243–50. 9389414 10.1007/s001250050816

[14] Yu PH DY. Endogenous formaldehyde as a potential factor of vulnerability of atherosclerosis: involvement of semicarbazide-sensitive amine oxidase-mediated methylamine turnover. Atherosclerosis. 1998;140:357–63. 9862279 10.1016/s0021-9150(98)00142-7

[15] Stolen CM, Madanat R, Marti L, Kari S, Yegutkin GG, Sariola H, et al Semicarbazide sensitive amine oxidase overexpression has dual consequences: insulin mimicry and diabetes-like complications. FASEB journal: official publication of the Federation of American Societies for Experimental Biology. 2004;18(6):702–4. Epub 2004/02/24. 10.1096/fj.03-0562fje . 14977883

[16] Yu PH ZD. Aminoguanidine inhibits semicarbazide-sensitive amine oxidase activity: implications for advanced glycation and diabetic complications. Diabetologia. 1997;40:1243–50. 9389414 10.1007/s001250050816

[17] Li HY WJ, Lin MS, Smith DJ, Vainio J, Lin CH, Chiang FT, Shih SR, Huang CH, Wu MY, Hsein YC, Chuang LM. Serum vascular adhesion protein-1 is increased in acute and chronic hyperglycemia. Clin Chim Acta. 2009;404:149–53. 10.1016/j.cca.2009.03.041 19336232

[18] Li HY JY, Chang TJ, Wei JN, Lin MS, Lin CH, Chiang FT, Shih SR, Hung CS, Hua CH, Smith DJ, Vanio J, Chuang LM. Serum vascular adhesion protein-1 predicts 10-year cardiovascular and cancer mortality in individuals with type 2 diabetes. Diabetes. 2011;60:993–9. 10.2337/db10-0607 21282368 PMC3046860

[19] Lin MS LH, Wei JN, Lin CH, Smith DJ, Vainio J, Shih SR, Chen YH, Lin LC, Kao HL, Chuang LM, Chen MF. Serum vascular adhesion protein-1 is higher in subjects with early stages of chronic kidney disease. Clin Biochem. 2008;41:1362–7. 10.1016/j.clinbiochem.2008.06.019 18644360

[20] Levey AS, Stevens LA, Schmid CH, Zhang YL, Castro AF 3rd, Feldman HI, et al A new equation to estimate glomerular filtration rate. Annals of internal medicine. 2009;150(9):604–12. Epub 2009/05/06. 19414839 10.7326/0003-4819-150-9-200905050-00006 PMC2763564

[21] Peripheral arterial disease in people with diabetes. Diabetes care. 2003;26(12):3333–41. Epub 2003/11/25. . 14633825 10.2337/diacare.26.12.3333

[22] Wu VC, Huang TM, Lai CF, Shiao CC, Lin YF, Chu TS, et al Acute-on-chronic kidney injury at hospital discharge is associated with long-term dialysis and mortality. Kidney international. 2011;80(11):1222–30. Epub 2011/08/13. 10.1038/ki.2011.259 . 21832983

[23] Boomsma F, Bhaggoe UM, van der Houwen AM, van den Meiracker AH. Plasma semicarbazide-sensitive amine oxidase in human (patho)physiology. Biochim Biophys Acta. 2003;1647:48–54. 12686107 10.1016/s1570-9639(03)00047-5

[24] Li HY LM, Wei JN, Hung CS, Chiang FT, Lin CH, Hsu HC, Su CY, Wu MY, Smith DJ, Vainio J, Chen MF, Chuang LM. Change of serum vascular adhesion protein-1 after glucose loading correlates to carotid intima-medial thickness in non-diabetic subjects. Clin Chim Acta 2009;403::97–101. 10.1016/j.cca.2009.01.027 19361461

[25] Kohavi R. A study of cross-validation and bootstrap for accuracy estimation and model selection. Proceedings of the Fourteenth International Joint Conference on Artificial Intelligence (San Mateo, CA: Morgan Kaufmann). 1995;2(12):1137–43.

[26] Brownlee M. The pathobiology of diabetic complications: a unifying mechanism. Diabetes. 2005;54:1615–25. 15919781 10.2337/diabetes.54.6.1615

[27] Venugopal SK, Devaraj S, Yuhanna I, Shaul P, Jialal I. Demonstration that C-reactive protein decreases eNOS expression and bioactivity in human aortic endothelial cells. Circulation. 2002;106(12):1439–41. Epub 2002/09/18. . 12234944 10.1161/01.cir.0000033116.22237.f9

[28] Streja D, Cressey P, Rabkin SW. Associations between inflammatory markers, traditional risk factors, and complications in patients with type 2 diabetes mellitus. Journal of diabetes and its complications. 2003;17(3):120–7. Epub 2003/05/10. . 12738395 10.1016/s1056-8727(02)00204-0

[29] Dalla Vestra M, Mussap M, Gallina P, Bruseghin M, Cernigoi AM, Saller A, et al Acute-phase markers of inflammation and glomerular structure in patients with type 2 diabetes. Journal of the American Society of Nephrology: JASN. 2005;16 Suppl 1:S78–82. Epub 2005/06/07. . 15938041 10.1681/asn.2004110961

[30] Giacco F, Brownlee M. Oxidative stress and diabetic complications. Circ Res. 2010;107:1058–70. 10.1161/CIRCRESAHA.110.223545 21030723 PMC2996922

[31] Yu PH, Deng YL. Endogenous formaldehyde as a potential factor of vulnerability of atherosclerosis: involvement of semicarbazide-sensitive amine oxidase-mediated methylamine turnover. Atherosclerosis. 1998;140(2):357–63. Epub 1998/12/23. . 9862279 10.1016/s0021-9150(98)00142-7

[32] Murata M NK, Fukuhara J, Kanda A, Kase S, Saito W, Ozawa Y, Mochizuki S, Kimura S, Mashima Y, Okada Y, Ishida S. Soluble vascular adhesion protein-1 accumulates in proliferative diabetic retinopathy. Invest Ophthalmol Vis Sci. 2012;53:4055–62. 10.1167/iovs.12-9857 22618595

[33] Aalto K, Maksimow M, Juonala M, Viikari J, Jula A, Kahonen M, et al Soluble vascular adhesion protein-1 correlates with cardiovascular risk factors and early atherosclerotic manifestations. Arteriosclerosis, thrombosis, and vascular biology. 2012;32(2):523–32. Epub 2011/11/26. 10.1161/atvbaha.111.238030 . 22116093

[34] Taal MW. Progress in risk prediction for people with chronic kidney disease. Current opinion in nephrology and hypertension. 2014;23(6):519–24. Epub 2014/09/17. 10.1097/mnh.0000000000000072 . 25226275

[35] Fernandes-Freitas I, Owen BM. Metabolic roles of endocrine fibroblast growth factors. Current opinion in pharmacology. 2015;25:30–5. Epub 2015/11/05. 10.1016/j.coph.2015.09.014 . 26531325

[36] Schnedl C, Fahrleitner-Pammer A, Pietschmann P, Amrein K. FGF23 in Acute and Chronic Illness. Disease markers. 2015;2015:358086 Epub 2015/10/23. 10.1155/2015/358086 26491212 PMC4600945

[37] Isakova T, Xie H, Yang W, Xie D, Anderson AH, Scialla J, et al Fibroblast growth factor 23 and risks of mortality and end-stage renal disease in patients with chronic kidney disease. JAMA: the journal of the American Medical Association. 2011;305(23):2432–9. Epub 2011/06/16. 10.1001/jama.2011.826 21673295 PMC3124770

[38] Kendrick J, Cheung AK, Kaufman JS, Greene T, Roberts WL, Smits G, et al FGF-23 associates with death, cardiovascular events, and initiation of chronic dialysis. Journal of the American Society of Nephrology: JASN. 2011;22(10):1913–22. Epub 2011/09/10. 10.1681/asn.2010121224 21903574 PMC3187186

[39] Wang C, Li C, Gong W, Lou T. New urinary biomarkers for diabetic kidney disease. Biomarker research. 2013;1(1):9 Epub 2013/11/21. 10.1186/2050-7771-1-9 24252392 PMC4177619

[40] Lee MJ, Jung CH, Kang YM, Jang JE, Leem J, Park JY, et al Serum Ceruloplasmin Level as a Predictor for the Progression of Diabetic Nephropathy in Korean Men with Type 2 Diabetes Mellitus. Diabetes & metabolism journal. 2015;39(3):230–9. Epub 2015/07/01. 10.4093/dmj.2015.39.3.230 26124993 PMC4483608

[41] Narita T, Hosoba M, Kakei M, Ito S. Increased urinary excretions of immunoglobulin g, ceruloplasmin, and transferrin predict development of microalbuminuria in patients with type 2 diabetes. Diabetes care. 2006;29(1):142–4. Epub 2005/12/24. . 16373913 10.2337/diacare.29.1.142

[42] Dunkel P, Gelain A, Barlocco D, Haider N, Gyires K, Sperlagh B, et al Semicarbazide-sensitive amine oxidase/vascular adhesion protein 1: recent developments concerning substrates and inhibitors of a promising therapeutic target. Current medicinal chemistry. 2008;15(18):1827–39. Epub 2008/08/12. . 18691041 10.2174/092986708785133022

